# Supplementary material for: Genetic evidence for assortative mating on alcohol consumption in the UK Biobank
Source: Nat Commun. 2019 Nov 19;10:5039. doi: 10.1038/s41467-019-12424-x (PMC6864067; doi:10.1038/s41467-019-12424-x)
Supplement: Supplementary file 1 — Supplementary Information [file 41467_2019_12424_MOESM1_ESM.pdf]

Supplementary material for "Genetic evidence for assortative mating on alcohol consumption in the UK Biobank"

LJ Howe et al

Supplementary Methods

Assortment: theory and simulations

Assuming a population of unrelated individuals (1000 males M and 1000 females F) where a phenotype P is assorted on (individuals more phenotypically similar for P are more likely to pair-up).

If $P \sim N(0, x)$ and is influenced by genetic factors $G \sim N(0, y)$ and non-genetic factors $E \sim N(0, x - y)$ such that:

(1) $\text{Cor}(G, P) = \sqrt{h^2}$ where h^2 is the proportion of variation in P explained by G.

It then follows that with assortment on P such that in each male female pair $\text{Cor}(P_M, P_F) = C$, that the expected spousal genotype-phenotype (GxP) and genetic correlation (GxG) associations capturing assortment are equivalent to:

(2) $\text{Cor}(G_M, P_F) = \text{Cor}(P_M, G_F) = C * \sqrt{h^2}$

(3) $\text{Cor}(G_M, G_F) = \frac{\text{Cov}(G_M, G_F)}{\sigma_{G_M}\sigma_{G_F}} = \text{C} * h^2$

Mendelian randomization analysis involves rescaling the spousal GxP association by the GxP association in the index individual and provides an unbiased estimate of assortment:

(4) $\text{MR}(P_M \rightarrow P_F) = \frac{\text{Cor}(G_M, P_F)}{\text{Cor}(G_M, P_M)} = \frac{\text{C}*\sqrt{h^2}}{\sqrt{h^2}} = C$

We used simulated data to confirm that the *GxP* and *GxG* estimates are influenced by the heritability (h^2) of the trait and the degree of phenotypic assortment C. We used a range of parameters: (h^2: 0.05, 0.1, 0.15, 0.2, 0.3, 0.4, 0.5) & (C: 0.1, 0.2, 0.3, 0.4, 0.5).

1000 simulations were run for each model with the mean correlation estimate reported and these results were consistent with the derived formulae.

Simulation results:

**Heritability of P**	**Degree of assortment: Phenotypic correlation C**	**Correlation (GxG, GxP): Mean of 1000 simulations**
0.05	0.1	0.005, 0.023
	0.2	0.008, 0.046
	0.3	0.015, 0.067
	0.4	0.021, 0.090
	0.5	0.026, 0.110
0.1	0.1	0.010, 0.033
	0.2	0.020, 0.062
	0.3	0.030, 0.092
	0.4	0.039, 0.126
	0.5	0.050, 0.157
0.15	0.1	0.015, 0.038
	0.2	0.029, 0.078
	0.3	0.045, 0.116
	0.4	0.059, 0.156
	0.5	0.074, 0.194
0.2	0.1	0.021, 0.046
	0.2	0.040, 0.089
	0.3	0.060, 0.133
	0.4	0.079, 0.177
	0.5	0.101, 0.225
0.3	0.1	0.030, 0.054
	0.2	0.059, 0.108
	0.3	0.088, 0.164
	0.4	0.121, 0.220
	0.5	0.150, 0.275
0.4	0.1	0.040, 0.064
	0.2	0.078, 0.127
	0.3	0.119, 0.190
	0.4	0.158, 0.251
	0.5	0.198, 0.315
0.5	0.1	0.049, 0.070
	0.2	0.099, 0.141
	0.3	0.149, 0.212
	0.4	0.201, 0.282
	0.5	0.250, 0.353

Supplementary Table 1: Association of rs1229984 with genetic principal components and birth coordinates

	**CC (%)**	**TC (%)**	**TT (%)**	**Hardy-Weinberg:** Chi^2, P-value
**385,287 individuals of European descent**	364,520 (94.6%)	20,194 (5.2%)	573 (0.1%)	274.7, $<10^{-16}$
**337,114 individuals of White British descent**	322,183 (95.6%)	14,743 (4.4%)	188 (<0.1%)	2.0, 0.16

Supplementary Table 2: Association of rs1229984 with genetic principal components and birth coordinates

**Principal components**	**385,287 individuals of European descent**		**337,114 individuals of White British descent**	
	**P-value**		**P-value**	
PC1	$<10^{-16}$		$<10^{-16}$	
PC2	$<10^{-16}$		$<10^{-16}$	
PC3	$<10^{-16}$		0.90	
PC4	$<10^{-16}$		$<10^{-16}$	
PC5	0.07		$<10^{-16}$	
PC6	0.04		0.00022	
PC7	$<10^{-16}$		0.074	
PC8	0.07		0.11	
PC9	0.86		0.11	
PC10	$1.53x10^{-9}$		0.00070	
**Birth-coordinates (units)**	**Beta (95% C.I.)[1]**	**P-value**	**Beta (95% C.I.)[1]**	**P-value**
North-South Axis (km north)	24.6 (22.2, 27.0)	$<10^{-16}$	19.4 (16.7, 22.0)	$<10^{-16}$
East-West Axis (km east)	-13.3 (-14.5, -12.1)	$<10^{-16}$	-10.3 (-11.7, -9.0)	$<10^{-16}$

1 Per additional major allele (associated with increased alcohol consumption)

Supplementary Table 3: Association of self-reported weekly alcohol consumption with genetic principal components and birth coordinates

**Principal components**	**385,287 individuals of European descent**		**337,114 individuals of White British descent**	
	**P-value**		**P-value**	
PC1	$<10^{-16}$		$3.3\text{x}10^{-11}$	
PC2	$<10^{-16}$		0.57	
PC3	$<10^{-16}$		$3.2\text{x}10^{-6}$	
PC4	$<10^{-16}$		$<10^{-16}$	
PC5	$<10^{-16}$		$<10^{-16}$	
PC6	$<10^{-16}$		0.36	
PC7	0.18		$1.1\text{x}10^{-5}$	
PC8	$2.2\text{x}10^{-10}$		0.0094	
PC9	$<10^{-16}$		$2.9\text{x}10^{-10}$	
PC10	$<10^{-16}$		$1.8\text{x}10^{-12}$	
**Birth-coordinates (units)**	**Beta (95% C.I.)**[1]	**P-value**	**Beta (95% C.I.)**[1]	**P-value**
North-South Axis (kilometres north)	0.19 (0.16, 0.22)	$<10^{-16}$	0.18 (0.15, 0.21)	$<10^{-16}$
East-West Axis (kilometres east)	-0.03 (-0.04, -0.02)	$7.1\text{x}10^{-5}$	-0.03 (-0.04, -0.01)	$4.4\text{x}10^{-4}$

1 Per 1 unit increase in weekly alcohol consumption

Supplementary Table 4: Spousal birth proximity and association with birth coordinates and principal components

	Complete spouse sample (N~47,549)				Spouses born within 100km (N~28,580)				Spouses born more than 100km apart (N~13,770)			
	**Spouse alcohol consumption difference per week (units)**		**Spousal rs1229984 genotype differences**		**Spouse alcohol consumption difference per week (units)**		**Spousal rs1229984 genotype differences**		**Spouse alcohol consumption difference per week (units)**		**Spousal rs1229984 genotype differences**	
**Spousal principal components difference**	**P-value**		**P-value**		**P-value**		**P-value**		**P-value**		**P-value**	
PC1	$1.22x10^{-8}$		$<10^{-16}$		0.007		$<10^{-16}$		0.003		$<10^{-16}$	
PC2	$2.28x10^{-7}$		$<10^{-16}$		0.49		$<10^{-16}$		$1.3x10^{-4}$		$<10^{-16}$	
PC3	$<10^{-16}$		$<10^{-16}$		$3.09x10^{-5}$		$<10^{-16}$		$7.63x10^{-13}$		$<10^{-16}$	
PC4	$<10^{-16}$		$<10^{-16}$		$4.72x10^{-16}$		$<10^{-16}$		$8.29x10^{-16}$		$<10^{-16}$	
PC5	$9.69x10^{-13}$		0.70		$4.89x10^{-12}$		0.14		$5.93x10^{-4}$		0.57	
PC6	0.27		$6.88x10^{-5}$		0.12		0.010		0.14		$2.37x10^{-4}$	
PC7	0.60		$<10^{-16}$		0.97		$<10^{-16}$		0.37		$<10^{-16}$	
PC8	0.12		0.15		0.031		0.10		0.69		$3.57x10^{-7}$	
PC9	0.15		0.90		0.13		0.71		0.16		0.98	
PC10	0.037		0.10		0.55		0.082		0.0027		0.23	
**Spousal Birth-coordinates difference (units)**	**Beta (95% C.I.)[1]**	**P-value**	**Beta (95% C.I.)[2]**	**P-value**	**Beta (95% C.I.)[1]**	**P-value**	**Beta (95% C.I.)[2]**	**P-value**	**Beta (95% C.I.)[1]**	**P-value**	**Beta (95% C.I.)[2]**	**P-value**
North-South Axis (kilometres north)	0.18 (0.10, 0.25)	$1.3x10^{-6}$	9.3 (5.2, 13.4)	$9.4x10^{-6}$	-0.003 (-0.017, 0.010)	0.62	0.46 (-0.33, 1.26)	0.25	0.56 (0.33, 0.78)	$1.2x10^{-6}$	25.7 (13.6, 37.8)	$3.0x10^{-5}$
East-West Axis (kilometres east)	-0.05 (-0.09, -0.01)	0.016	-4.1 (1.7, 6.4)	$7.5x10^{-4}$	-0.000 (-0.014, 0.013)	0.95	-0.75 (-1.58, 0.08)	0.078	-0.15 (-0.28, -0.03)	0.019	-10.2 (-3.4, -17.1)	0.0034

1 Per 1 unit increase in weekly alcohol consumption

2 Per additional major allele (associated with increased alcohol consumption)

Supplementary Table 5: Comparison of Mendelian randomization and genotypic concordance estimates between spouse-pair samples stratified on birth proximity

**Mendelian randomization estimates:** unit increase in partner's alcohol consumption per 1-unit increase in weekly alcohol consumption in index individual (95% C.I.)			**Genotypic concordance estimates:** increase in number of major alleles in partner per 1 major allele increase in index individual (95% C.I.)		
**Complete spouse sample (N~47,549)**	**Spouses born within 100km (N~28,580)**	**Spouses born more than 100km apart (N~13,770)**	**Complete spouse sample (N~47,549)**	**Spouses born within 100km (N~28,580)**	**Spouses born more than 100km apart (N~13,770)**
0.26 (0.15, 0.38)	0.29 (0.15, 0.44)	0.19 (-0.01, 0.40)	0.0019 (0.010, 0.028)	0.017 (0.005, 0.029)	0.009 (-0.008, 0.026)

Supplementary Table 6: Information on alcohol questionnaire variables

Alcohol variable	Median (Q1, Q3); Max
Alcoholic units a week (N=95,059)[1]:	12 (1, 24); 312
Spirit measures	0 (0,1); 200
Glasses of white wine	0 (0,2); 80
Glasses of red wine	1 (0,4); 90
Glasses of fortified wine	0 (0,0); 84
Pints of beer or cider	0 (0,2); 72
Current drinking status (N= 95,059):	
Never: N (%)	2669 (2.8%)
Previous: N (%)	2630 (2.8%)
Current: N (%)	89760 (94.4%)
Current alcohol intake frequency (N= 95,059):	
Never: N (%)	5299 (5.6%)
Special occasions only: N (%)	8583 (9.0%)
One to three times a month: N (%)	9497 (10.0%)
Once or twice a week: N (%)	25253 (26.6%)
Three or four times a week: N (%)	25222 (26.5%)
Daily or almost daily: N (%)	21205 (22.3%)

1 Self-report non or former drinkers had values imputed to 0 for relevant variables.

Supplementary Table 7: Weekly alcohol consumption per rs1229984 genotype

	385,287 individuals of European descent			337,114 individuals of White British descent		
	**CC (N=363,036) Median (Q1, Q3)**	**TC (N=20,146) Median (Q1, Q3)**	**TT (N=569) Median (Q1, Q3)**	**CC (N=320,699) Median (Q1, Q3)**	**TC (N=14,728) Median (Q1, Q3)**	**TT (N=188) Median (Q1, Q3)**
**Weekly alcohol consumption (units)**	12.0 (0, 24)	7.0 (0, 17.5)	2.0 (0, 13)	12.0 (0, 24.0)	8.0 (0, 18)	4.3 (0, 18.1)

Supplementary Figure 1: Subsets of UK Biobank utilised in analyses

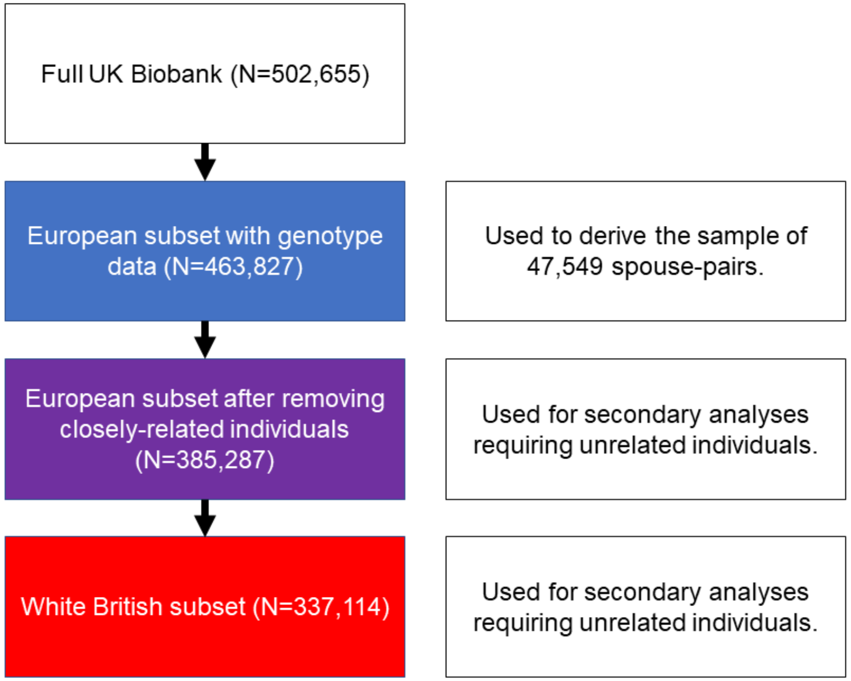

A flowchart illustrating the different subsets of UK Biobank used in analyses and how each subset was constructed.
